# One-Pot High-Current-Density Electrodeposition of Ni–Ce and Ni–Fe–Ce Catalysts on Bamboo-Derived Carbon Fabric for Alkaline Water Splitting

**DOI:** 10.3390/molecules31172969

**Published:** 2026-08-25

**Authors:** Sun-Woo Lee, Sunghoon Ahn

**Affiliations:** Department of Biochemical Engineering, Chosun University, Gwangju 61452, Republic of Korea

**Keywords:** alkaline water electrolysis, hydrogen evolution reaction, oxygen evolution reaction, electrodeposition, cerium oxide, NiFe catalyst, biomass-derived carbon, self-supported electrode

## Abstract

Self-supported electrodes that combine high activity, durability, and low-cost manufacturing are essential for scalable alkaline water electrolysis. Here, we report a one-pot, high-current-density electrodeposition platform that grows Ni-rich bimetallic catalysts directly on a carbon fabric derived from a mass-produced bamboo-cellulose kitchen wipe (bamboo-derived carbon fabric, BCF). Using a single NiCl_2_/NH_4_Cl base bath containing 5 mM of a selectable secondary metal ion (Ce, Fe, W, or Mo), galvanostatic deposition at 1 A cm^−2^ for 15 min produces conformal polycrystalline catalyst shells on the individual carbon fibers. The Ce-containing cathode (BCF@NiCe) delivers hydrogen evolution overpotentials of 96.8 mV at 20 mA cm^−2^ and 222 mV at 1 A cm^−2^, rivaling a Pt/C benchmark on the same substrate at industrially relevant current densities, which is attributed to the cooperative interface between metallic Ni and nanocrystalline, oxygen-vacancy-rich CeO_2−x_ together with a superhydrophilic fibrous architecture that releases fine H_2_ microbubbles. Adding Fe to the same bath yields a Ni–Fe–Ce anode (BCF@NiFeCe) that outperforms a RuO_2_ benchmark for oxygen evolution above 0.1 A cm^−2^ (η = 312 mV at 0.1 A cm^−2^) with a Tafel slope of 60 mV dec^−1^. Both electrodes operate stably for 200 h of continuous electrolysis, with the Ni/CeO_2−x_ nanostructure, the oxygen-vacancy population, and the surface chemical states fully preserved after the test, and the same protocol extends to Ni–W and Ni–Mo on nickel foam, establishing a versatile, low-cost route to high-current-density electrodes for green hydrogen production.

## 1. Introduction

Green hydrogen produced by water electrolysis powered by renewable electricity is a cornerstone of the transition to a carbon-neutral energy system [1,2]. Alkaline water electrolysis (AWE) and the emerging anion exchange membrane water electrolysis (AEMWE) permit the use of platinum-group-metal (PGM)-free catalysts based on earth-abundant transition metals, and decades of catalyst research have produced Ni-based materials with excellent intrinsic activity for both the hydrogen evolution reaction (HER) and the oxygen evolution reaction (OER) [3,4,5]. However, most laboratory studies evaluate powder catalysts sprayed onto current collectors with polymeric binders and at current densities below 100 mA cm^−2^, whereas practical electrolyzers operate at 0.5–2 A cm^−2^ [1,6]. At such current densities, sprayed catalyst layers suffer from weak adhesion, binder degradation, and delamination driven by vigorous gas evolution, so that electrodes combining high activity with mechanical robustness at ampere-level current densities remain scarce [6,7].

Self-supported electrodes, in which the catalyst is grown directly on a conductive substrate, are an effective answer to these interface problems, offering intimate electrical contact and binder-free adhesion [8]. Among the available growth methods, electrodeposition is particularly attractive for scale-up because it is a room-temperature, atmospheric-pressure process whose loading and morphology are controlled simply by charge and current. Recently, Serban et al. reported a distinctive electrodeposition chemistry in which a high concentration of NH_4_Cl serves as the sole supporting electrolyte, without any pH buffer or complexing agent; the anodic chlorine evolution and its subsequent dissolution keeps the bath pH from rising, enabling deposition current densities an order of magnitude higher than conventional Ni plating baths and yielding a compact, strongly adherent NiMo catalyst with record AEMWE performance [9]. This chemistry, however, has so far been demonstrated only for the Ni–Mo couple on commercial gas diffusion substrates, and its generality with respect to both the secondary metal and the substrate is unexplored.

Two extensions of this platform are especially appealing. The first concerns the substrate. Commercial carbon papers and metallic meshes dominate self-supported electrode research, but biomass-derived carbon textiles offer a sustainable and remarkably inexpensive alternative: cellulose fabrics are mass-produced consumer goods, and their carbonization yields freestanding, flexible conductive fabrics that retain the woven fiber network [10,11]. The second concerns the choice of the secondary metal. Cerium cannot be electrodeposited as a metal from aqueous solution because of its strongly negative reduction potential (E°(Ce^3+^/Ce) ≈ −2.34 V vs. SHE) [12]. Under a high cathodic current, however, the hydrogen evolution accompanying Ni deposition raises the local pH at the electrode surface, so that Ce^3+^ precipitates as hydroxide/oxide species that are continuously buried by the growing Ni matrix. High-current deposition thus converts the interfacial alkalization—an effect that the NH_4_Cl chemistry suppresses in the bulk bath—into a synthetic tool that embeds CeO_x_ within a metallic Ni shell in a single step. The resulting Ni/CeO_x_ interfaces are highly desirable: oxygen vacancies associated with the Ce^3+^/Ce^4+^ redox couple promote water dissociation, the rate-limiting Volmer step of alkaline HER on metallic Ni [13,14,15], while for the OER, Ce has been shown to act as a redox mediator and stabilizer in Ni–Fe (oxy)hydroxide catalysts, the state-of-the-art PGM-free OER system [16,17].

**Figure 1:**
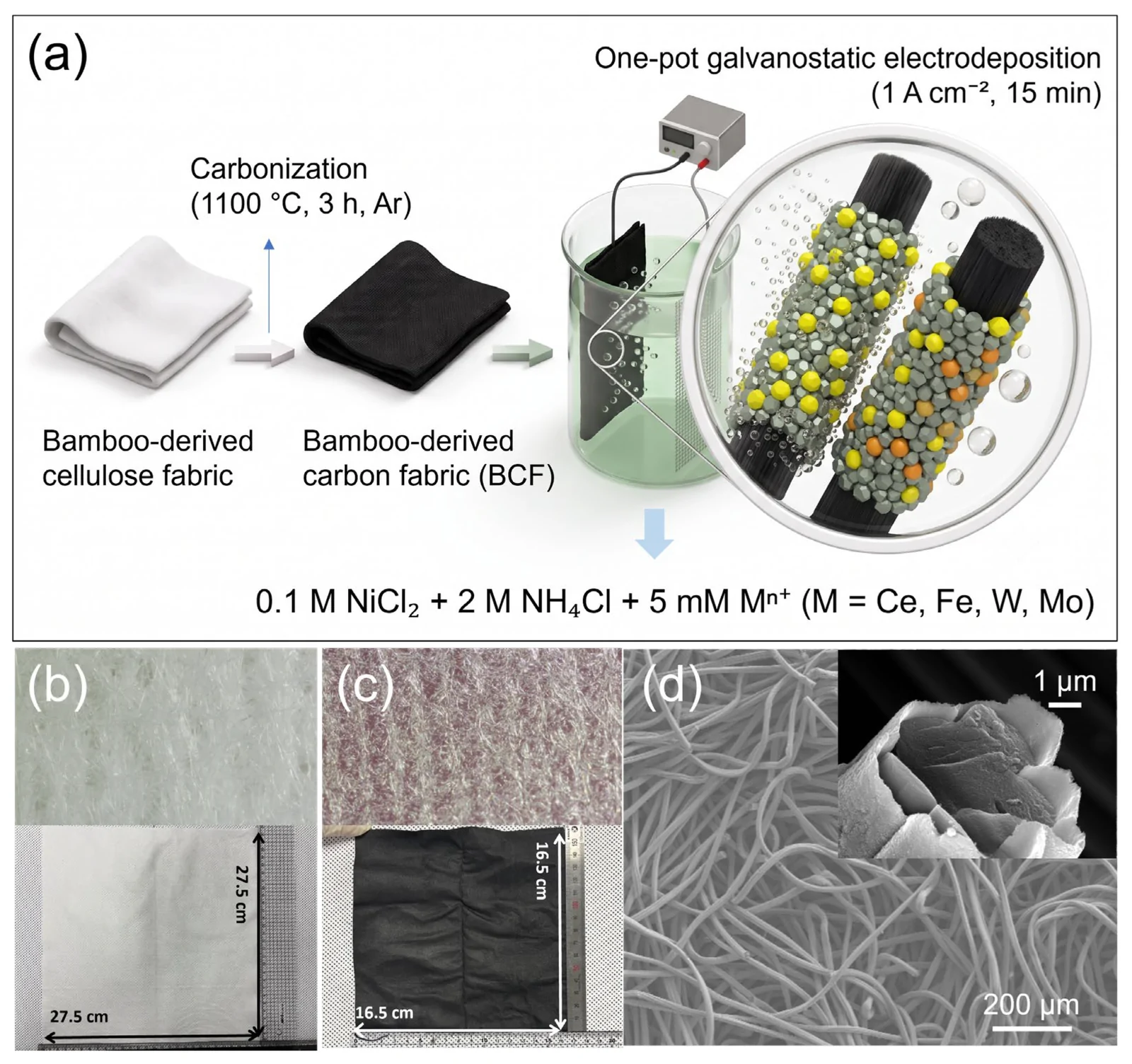
(**a**) Schematic illustration of the fabrication of BCF-supported Ni–M catalyst electrodes via one-pot high-current-density electrodeposition. (**b**,**c**) Photographs of the bamboo-cellulose fabric before (**b**) and after (**c**) carbonization. (**d**) SEM image of BCF@Ni (inset: fracture cross-section showing the carbon-fiber core and the plated Ni shell).

Here we combine these two extensions into a single, general electrode-fabrication platform (Figure 1). A carbon fabric (BCF) is obtained by carbonizing a commercial bamboo-cellulose kitchen wipe, and Ni-rich bimetallic catalysts are grown on it by one-pot galvanostatic electrodeposition at 1 A cm^−2^ from a common 0.1 M NiCl_2_/2 M NH_4_Cl bath containing 5 mM of a selectable secondary metal ion. With Ce^3+^ in the bath, the resulting BCF@NiCe is a high-performance HER cathode whose activity approaches a Pt/C benchmark on the same substrate and surpasses it at ampere-level current densities, aided by a superhydrophilic fibrous architecture that promotes the release of fine gas microbubbles. With Ce^3+^ and Fe^3+^ together, the deposit crystallizes as a NiFe alloy decorated with CeO_x_, and the resulting BCF@NiFeCe outperforms a RuO_2_ benchmark for the OER at current densities above 0.1 A cm^−2^. Both electrodes sustain 200 h of continuous operation, and the identical protocol produces active Ni–W and Ni–Mo HER catalysts on nickel foam, demonstrating that the high-current NH_4_Cl chemistry is a general platform in which the practitioner simply selects the dissolved element. Given the negligible cost of the substrate and the simplicity of the DC-power-supply-driven process, this work outlines a practical route to durable, ampere-level electrodes for green hydrogen production.

## 2. Results and Discussion

### 2.1. Fabrication and Characterization of BCF@NiCe

The fabrication route is summarized in Figure 1a. Carbonization of the bamboo-cellulose fabric (27.5 × 27.5 cm^2^) at 1100 °C under Ar produced a freestanding, flexible carbon fabric of 16.5 × 16.5 cm^2^ (≈36% of the original area) that preserved the nonwoven fiber network of the pristine wipe (Figure 1b,c). To provide a plain nickel-surface reference on the same fabric—playing the role of the Ni foam or Ni felt substrates commonly used in alkaline electrolysis—the BCF was coated with a thin Ni layer by conventional low-current electroplating (0.25 A cm^−2^, 15 min) from the additive-free base bath, yielding BCF@Ni. Scanning electron microscopy (SEM) shows that the ~10 μm fibers remain individually separated within an open, entangled network after plating (Figure 1d), and the fracture cross-section (Figure 1d, inset) reveals the carbon-fiber core wrapped by a conformal metallic Ni shell, confirming that electrodeposition proceeds uniformly around each fiber throughout the three-dimensional fabric. This architecture—micrometer-scale conductive fibers with large open porosity—serves simultaneously as current collector and gas diffusion medium.

**Figure 2:**
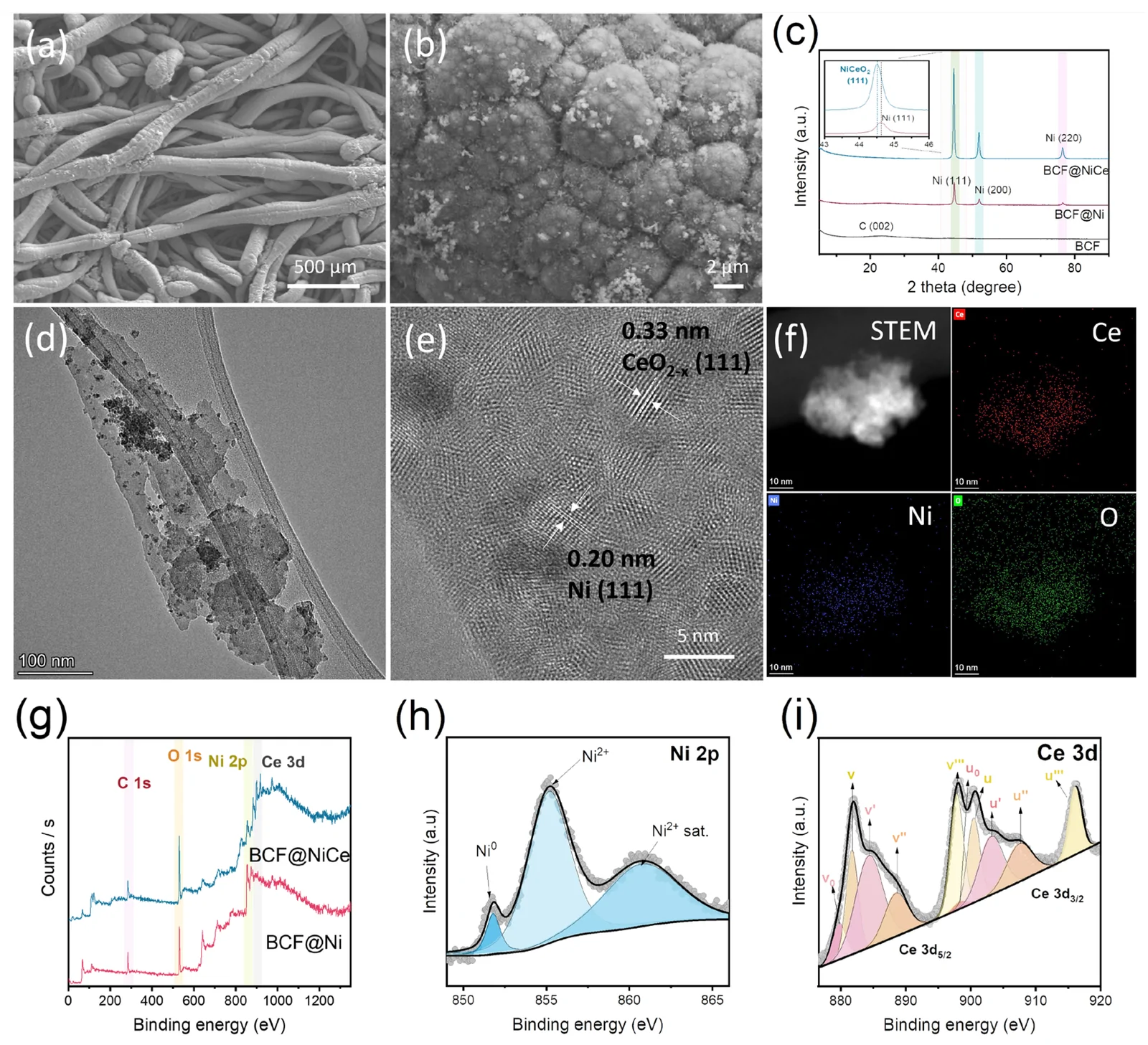
Characterization of BCF@NiCe: (**a**,**b**) SEM images at increasing magnification; (**c**) XRD patterns of BCF, BCF@Ni, and BCF@NiCe (inset: magnified region around the Ni(111) reflection); (**d**) low-magnification TEM image of the catalyst detached from the BCF support; (**e**) HRTEM image showing lattice fringes of 0.33 nm and 0.20 nm, assigned to the (111) planes of CeO_2−x_ (expanded relative to bulk CeO_2_, 0.312 nm) and metallic Ni, respectively; (**f**) HAADF-STEM image and the corresponding EDX elemental maps (Ce, Ni, O); (**g**) XPS survey spectra of BCF@Ni and BCF@NiCe; (**h**) high-resolution Ni 2p spectrum of BCF@NiCe; (**i**) Ce 3d spectrum of BCF@NiCe with deconvolution into Ce^3+^ and Ce^4+^ components.

Electrodeposition from the Ce-containing bath at the optimized condition (1 A cm^−2^, 15 min; Section 2.2) transformed the fabric into BCF@NiCe. The fiber network remains open after deposition (Figure 2a), while each fiber is individually wrapped by a continuous, cauliflower-like polycrystalline shell with secondary nanoparticle features dispersed over the micrometer-sized granular domains (Figure 2b and Figure S1). The Ni-plated control shows a similarly conformal but smoother shell, with only Ni and O detected in the energy-dispersive X-ray spectroscopy (EDX) maps (Figure S2). SEM/EDX mapping of a single NiCe-coated fiber (Figure S3) shows that Ni forms a dense conformal shell around the carbon core, with O and Ce distributed homogeneously throughout the shell, confirming that the Ce species are incorporated within the Ni matrix rather than segregated at the outer surface. To resolve the nanostructure of the shell, the catalyst was detached from the fabric and examined by transmission electron microscopy (TEM) (Figure 2d–f). The low-magnification image (Figure 2d) shows aggregates of the detached catalyst, and high-resolution TEM (HRTEM; Figure 2e) reveals a dense mosaic of nanocrystalline domains of 2–5 nm. Two distinct sets of lattice fringes coexist within single fields of view: spacings of 0.20 nm matching Ni(111), and spacings of 0.33 nm assigned to the (111) planes of CeO_2−x_. The latter is expanded by 3–5% relative to bulk CeO_2_ (0.312 nm), the established signature of nanocrystalline ceria containing a high density of Ce^3+^ and oxygen vacancies [18,19]. Nanoscale EDX mapping of the same aggregates (Figure 2f) shows Ce, Ni, and O intimately co-distributed, confirming that the CeO_2−x_ domains are dispersed throughout the metallic Ni matrix at the nanometer scale—precisely the intimate Ni/CeO_2−x_ contact required for the bifunctional alkaline HER mechanism. Area-averaged SEM-EDX gives a deposit composition of Ni:Ce = 96.1:3.9 wt%, i.e., a Ce fraction well below the 4.8 mol% Ce content of the bath, consistent with precipitation-mediated (rather than Faradaic) incorporation of Ce.

X-ray diffraction (XRD; Figure 2c) identifies the crystallographic consequence of Ce incorporation. Bare BCF shows only the broad C(002) reflection of disordered carbon. BCF@Ni exhibits the fcc Ni(111), (200), and (220) reflections, and BCF@NiCe shows the same metallic Ni framework together with a distinct shoulder at the low-angle side of the Ni(111) peak (inset), which, in light of the HRTEM analysis (Figure 2e), is assigned to reflections of the lattice-expanded nanocrystalline CeO_2−x_ phase rather than to a distortion of the Ni lattice [12]. The X-ray photoelectron spectroscopy (XPS) survey spectra (Figure 2g) confirm the presence of Ce 3d in BCF@NiCe, and the high-resolution Ni 2p spectrum of BCF@NiCe (Figure 2h; that of BCF@Ni is shown in Figure S4) is deconvoluted into metallic Ni^0^, Ni^2+^, and satellite components, indicating a metallic interior covered by a native (oxy)hydroxide surface, as expected for Ni electrodeposits handled in air; the smaller relative weight of the Ni^0^ component in BCF@NiCe (≈6% vs. ≈27% for BCF@Ni) reflects the additional CeO_x_-containing surface coverage. Most importantly, the Ce 3d spectrum (Figure 2i) displays the full v/u multiplet structure together with the v_0_/u_0_ components, evidencing coexisting Ce^3+^ and Ce^4+^ states [20]. The Ce^3+^ fraction of 35.6%, obtained from a ten-component deconvolution with the spin–orbit splitting and 3d_3/2_/3d_5/2_ area ratio constrained (see Section 3.4), corresponds to an appreciable population of oxygen vacancies in the CeO_x_ component, which is the structural feature widely credited with accelerating water dissociation at Ni/CeO_x_ interfaces [13,14,21].

### 2.2. Deposition Current Density Window

Because the central hypothesis of this work is that a high deposition current density is not merely tolerated but required by the bath chemistry, the deposition current density was varied systematically at 0.25, 0.5, 0.75, 1.0, 1.5, 2.0, and 3.0 A cm^−2^ at a fixed deposition time of 15 min. Under this fixed-time protocol, a higher current density simultaneously increases the deposition rate and the deposited amount, so the screening identifies the practically optimal fabrication condition.

**Figure 3:**
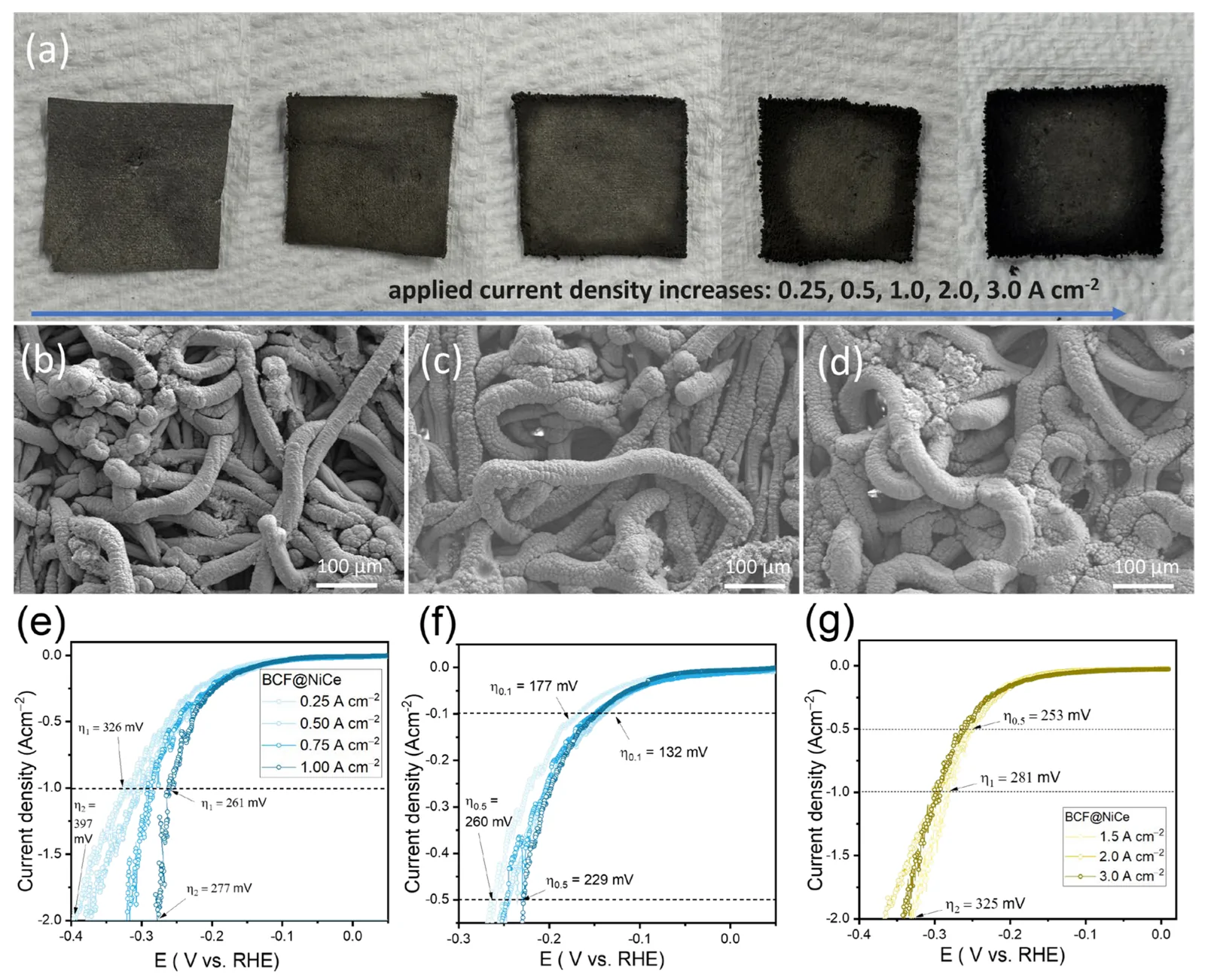
Optimization of the deposition current density: (**a**) photographs of BCF@NiCe deposited at 0.25–3.0 A cm^−2^ for 15 min; (**b**–**d**) SEM images of the deposits prepared at 1.0, 2.0, and 3.0 A cm^−2^, respectively; (**e**) HER linear sweep voltammetry (LSV) curves of the deposits prepared at 0.25–1.00 A cm^−2^ and (**f**) the corresponding low-current-density region; (**g**) LSV curves of the 1.5–3.0 A cm^−2^ deposits.

The photographs in Figure 3a already reveal a qualitative evolution: the fabric darkens progressively with increasing current density, and above 2.0 A cm^−2^ pronounced dendritic overgrowth appears along the electrode edges, indicating the onset of transport-limited deposition. SEM (Figure 3b–d) shows the corresponding morphological window: at low current density the fibers are only thinly and unevenly covered, at 1 A cm^−2^ the shells are continuous and conformal, and at higher current densities, the coating grows increasingly nodular and loosely packed. The HER activity tracks this window (Figure 3e,f): the overpotential at 0.1 A cm^−2^ decreases from 177 mV (0.25 A cm^−2^) to 132 mV at the optimum, and the overpotential at 1 A cm^−2^ reaches 261 mV. Increasing the deposition current density further to 1.5–3.0 A cm^−2^ degrades the performance again (η at 1 A cm^−2^ ≥ 281 mV; Figure 3g), consistent with the fragile dendritic morphology. The optimum of 1 A cm^−2^ for 15 min—a deposition current density four- to twenty-fold higher than conventional Ni plating practice [22]—was therefore adopted for all subsequent samples. We note that this optimum reproduces, on an entirely different substrate and with a different secondary metal, the compact-deposit rationale proposed for the NH_4_Cl chemistry [9], supporting its generality.

Three additional measurements consolidate this deposition picture. First, the working-electrode potential recorded during galvanostatic deposition (three-electrode configuration; Figure S5) settles within tens of seconds to a quasi-steady value at each current density and remains stable throughout the deposition, with no passivation-type potential excursion; together with the NH_4_^+^/NH_3_ buffering of the interfacial pH and the galvanostatic mode of operation, this rules out the hydroxide-mediated self-termination reported for potentiostatic deposition in dilute, unbuffered baths [23]. Second, gravimetric analysis at deposition times of 5, 15, and 30 min yields loadings of 31, 87, and 142 mg cm^−2^, i.e., a continuous, near-linear mass gain with no sign of saturation, corresponding to apparent current efficiencies of 34.0, 31.8, and 25.9% (Faraday’s law, two-electron Ni^2+^ reduction; the measured mass includes the co-deposited CeO_x_). The current efficiency of ~30% quantifies the intended partition of the applied current: the dominant HER sustains the interfacial alkalinization that drives Ce incorporation, while vigorous H_2_ evolution enhances convective Ni^2+^ transport far above the quiescent diffusion limit. The modest decline at 30 min is quantitatively consistent with progressive depletion of the 150 mL bath (14, 40, and 65% of the initial Ni^2+^ inventory consumed at 5, 15, and 30 min, respectively). Third, the deposition time and the Ce precursor concentration were varied around the optimum (Figure S6): 15 min gives the best HER activity among 5–60 min deposits, and baths containing 2.5 or 10 mM CeCl_3_ (BCF@NiCe-2.5 and BCF@NiCe-10) yield inferior activity to the standard 5 mM composition, confirming that the adopted condition is optimal with respect to deposition current density, time, and Ce concentration.

### 2.3. Hydrogen Evolution Performance of BCF@NiCe

**Figure 4:**
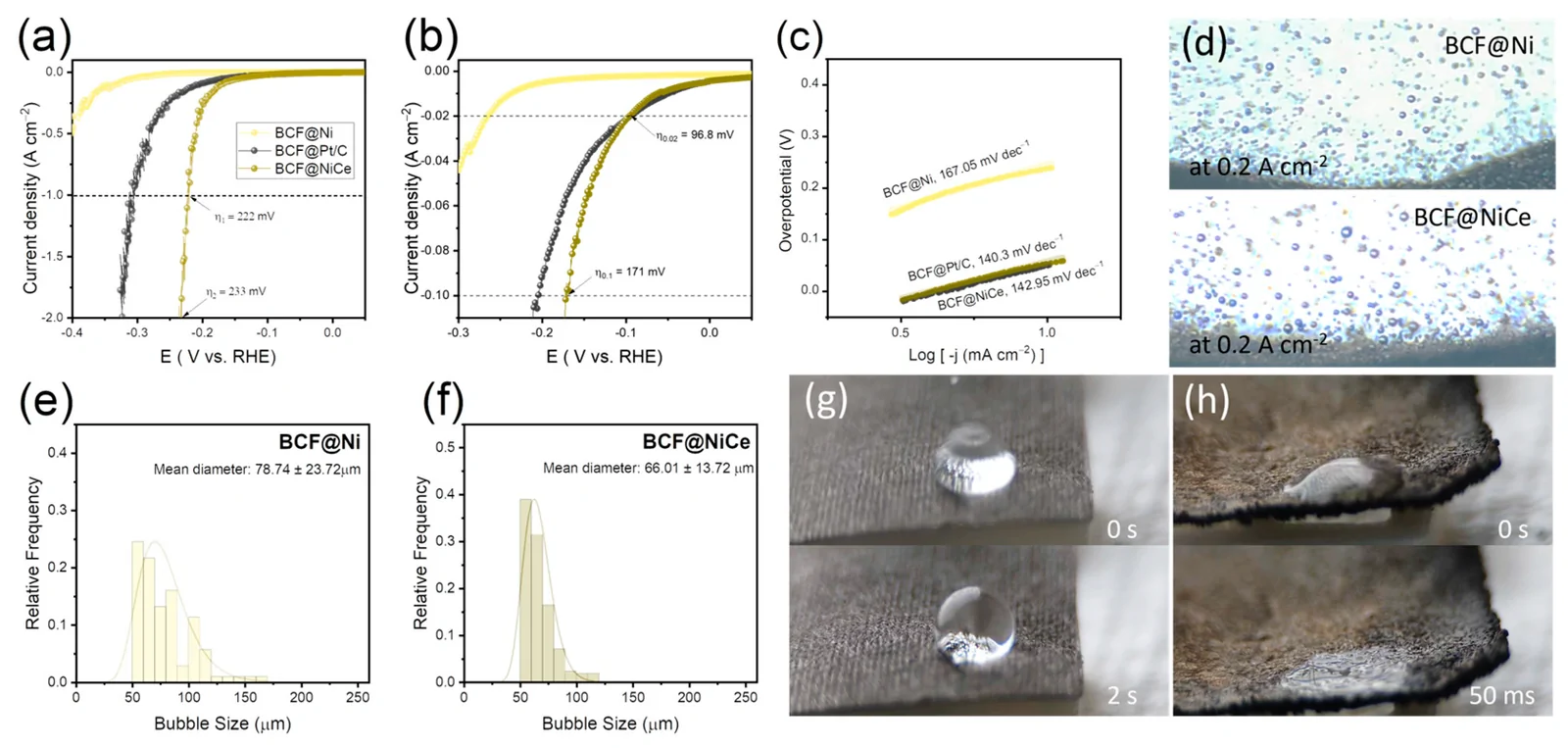
HER performance and bubble-release behavior of BCF@NiCe in 1 M KOH: (**a**) LSV curves of BCF@Ni, BCF@Pt/C, and BCF@NiCe; (**b**) low-current-density region; (**c**) Tafel plots; (**d**) in situ photographs of H_2_ bubble evolution on BCF@Ni and BCF@NiCe at 0.2 A cm^−2^; (**e**,**f**) near-surface bubble size distributions of BCF@Ni (**e**) and BCF@NiCe (**f**) obtained by quantitative image analysis; (**g**,**h**) water-droplet absorption snapshots on BCF@Ni (**g**) and BCF@NiCe (**h**).

Figure 4 benchmarks BCF@NiCe against the Ni-plated control (BCF@Ni), which represents a plain nickel surface analogous to conventional Ni foam or Ni felt substrates, and against a Pt/C catalyst sprayed on the same fabric (BCF@Pt/C), thereby isolating the contribution of the catalyst chemistry from that of the substrate. BCF@NiCe requires overpotentials of only 96.8 mV at 20 mA cm^−2^ and 171 mV at 0.1 A cm^−2^ (Figure 4b), and—decisively for practical operation—222 mV at 1 A cm^−2^ and 233 mV at 2 A cm^−2^ (Figure 4a). The comparison with the PGM benchmark mirrors the behavior reported for other high-performance Ni-based cathodes [9]: BCF@Pt/C is more active in the low-current-density regime, reflecting the higher intrinsic activity of Pt, but its polarization curve crosses that of BCF@NiCe below 1 A cm^−2^, beyond which the PGM-free electrode is superior. The Tafel slope of BCF@NiCe (143 mV dec^−1^) is essentially identical to that of BCF@Pt/C (140 mV dec^−1^) and markedly smaller than that of BCF@Ni (167 mV dec^−1^) (Figure 4c), and electrochemical impedance spectroscopy (EIS) shows the smallest charge-transfer resistance for BCF@NiCe (Figure S7). Together, these observations indicate that CeO_x_ incorporation accelerates the water-dissociation-limited alkaline HER kinetics on Ni, in line with the established bifunctional picture in which oxygen-vacancy-rich CeO_x_ dissociates water and supplies protons to adjacent Ni sites for hydrogen recombination [13,14,21]. To separate the surface-area and intrinsic contributions to this enhancement, the double-layer capacitances (C_dl_) were measured from scan-rate-dependent cyclic voltammograms (CVs) in the non-Faradaic region (Figure S8), giving C_dl_ = 1.10, 9.85, and 9.11 mF cm^−2^ for BCF@Ni, BCF@NiCe, and BCF@NiFeCe, respectively—i.e., electrochemically active surface areas (ECSAs) of 27.5, 246, and 228 cm^2^ (Cs = 40 µF cm^−2^ [24]) and a ~9-fold surface-area amplification by the Ce-assisted co-deposition. Crucially, the ECSA-normalized polarization curves (Figure S9) show that BCF@NiCe retains a lower HER overpotential than BCF@Ni even per unit real surface area (Δη ≈ 80 mV at 1 mA cm^−2^_ECSA_), demonstrating that the enhancement combines the ~9-fold ECSA gain with a genuine improvement of the intrinsic activity, consistent with the oxygen-vacancy-rich CeO_2−x_ identified above.

The advantage at ampere-level current densities has a second, macroscopic origin: gas management. In situ imaging of H_2_ evolution at 0.2 A cm^−2^ (Figure 4d) shows both electrodes releasing dense streams of microbubbles, and quantitative image analysis of the near-surface bubble populations (Figure 4e,f) yields mean area-equivalent diameters of 78.7 ± 23.7 µm for BCF@Ni and 66.1 ± 18.7 µm for BCF@NiCe. Both values are an order of magnitude smaller than the bubble sizes typically observed on planar Ni electrodes (hundreds of micrometers) [25], indicating that the fine-bubble release originates primarily from the fibrous BCF architecture, whose micrometer-scale fiber curvature limits bubble growth and adhesion, rather than from a catalyst-specific surface effect. Droplet-impingement snapshots (Figure 4g,h) show that a water droplet placed on BCF@Ni retains its shape for at least 2 s, whereas on BCF@NiCe it is completely imbibed within 50 ms. Such superhydrophilic behavior of the hierarchically rough NiCe shell further assists electrolyte penetration throughout the fiber network and the rapid detachment of evolved bubbles before they can blanket the surface [25]. At current densities where mass transport and bubble shielding dominate the overpotential, these gas-management characteristics compound the intrinsic kinetic gain, explaining why the polarization curve of BCF@NiCe remains nearly vertical up to 2 A cm^−2^.

### 2.4. Structural and Chemical Stability of BCF@NiCe Under Prolonged HER Operation

**Figure 5:**
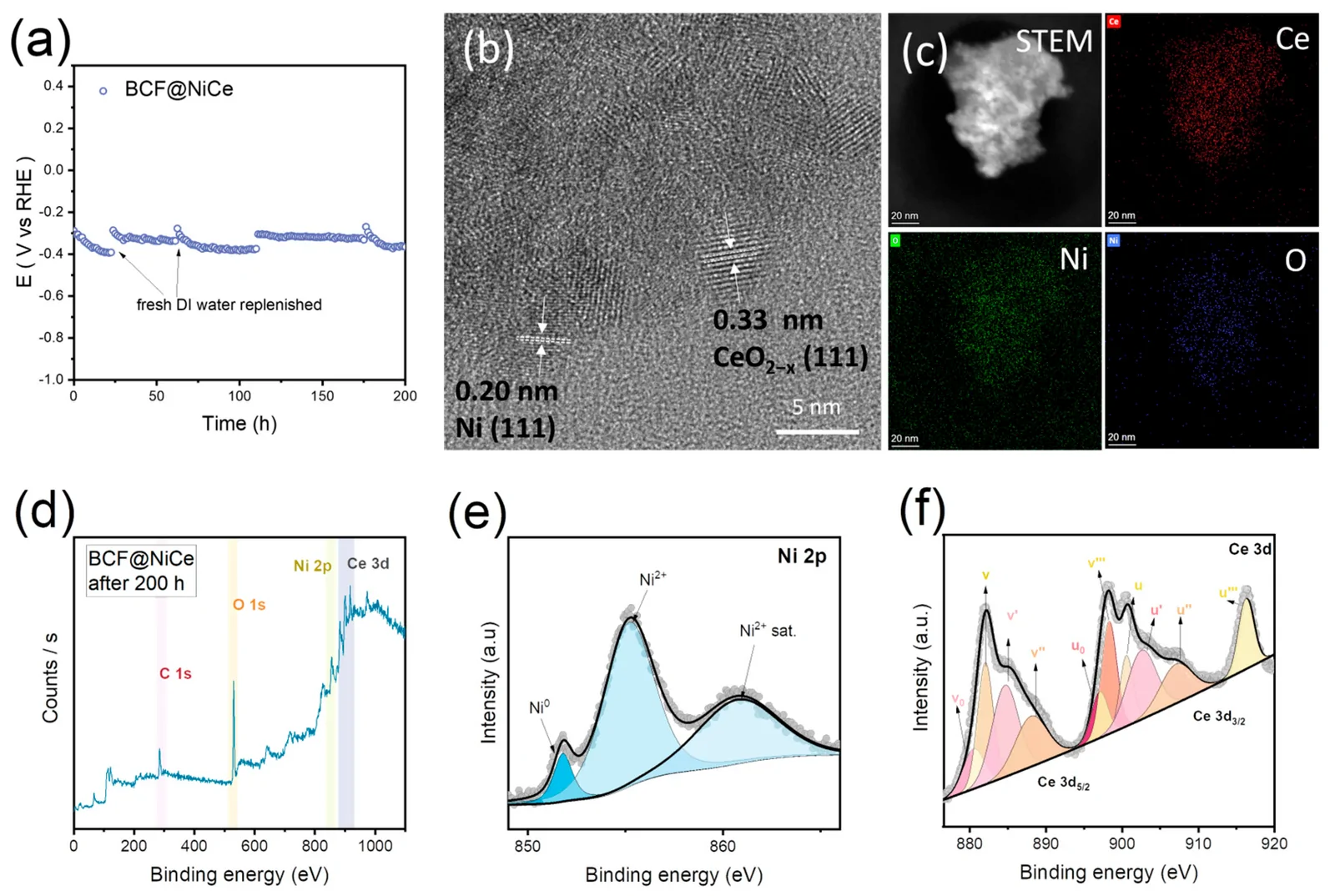
Structural and chemical stability of BCF@NiCe after the 200 h HER durability test: (**a**) chronopotentiometric profile at a constant current density of 1 A cm^−2^ in 1 M KOH for 200 h; (**b**) HRTEM image after the test, showing the preserved Ni(111) (0.20 nm) and CeO_2−x_(111) (0.33 nm) lattice fringes; (**c**) HAADF-STEM image and the corresponding EDX maps (Ce, Ni, O), confirming the retained Ni–Ce co-distribution; (**d**–**f**) XPS spectra after the test: (**d**) survey, (**e**) Ni 2p, and (**f**) Ce 3d, showing surface chemical states essentially identical to those of the as-prepared electrode (Ce^3+^ fraction of 38.0% vs. 35.6%).

The 200 h galvanostatic HER operation at 1 A cm^−2^ (Figure 5a) provides the first indication of stability: the potential remains essentially unchanged over the entire test, with the small periodic steps coinciding with the replenishment of deionized (DI) water to compensate electrolyte evaporation. To establish whether this electrochemical stability reflects true structural invariance, the electrode recovered after the 200 h galvanostatic test was subjected to full post-mortem characterization (Figure 5b–f and Figure S10). HRTEM of the post-test catalyst (Figure 5b) reveals the same nanocrystalline mosaic as the as-prepared material, with both the Ni(111) (0.20 nm) and the lattice-expanded CeO_2−x_(111) (0.33 nm) fringes preserved, and EDX mapping (Figure 5c) confirms the retained nanoscale Ni–Ce co-distribution; no Ce leaching or phase segregation is detectable. XPS (Figure 5d–f) corroborates this at the surface-chemistry level: the Ce^3+^ fraction after 200 h (38.0%) is identical within fitting uncertainty to the as-prepared value (35.6%), the surface Ni^2+^/Ni^0^ ratio is unchanged (93/7 vs. 94/6), and all peak positions are preserved. The oxygen-vacancy population that underpins the HER activity is therefore not a transient feature of the fresh catalyst but is retained under prolonged cathodic operation, and SEM after the test shows the deposit morphology and adhesion intact (Figure S10).

The mechanical robustness implied by these observations was probed directly by an accelerated ultrasonic delamination test (Figure S11). After 60 min of sonication in deionized water—cavitation shear far exceeding the bubble-induced forces of normal operation—the directly electrodeposited BCF@NiCe remains visually intact and its solution clear, whereas an identically treated binder-coated control (Pt/C + Nafion on the same BCF) sheds its catalyst massively, blackening the solution. Together with the 200 h operation under vigorous gas evolution, this demonstrates that the high-current-grown shells satisfy the mechanical demands of practical service without any binder.

### 2.5. Oxygen Evolution Performance of BCF@NiFeCe

**Figure 6:**
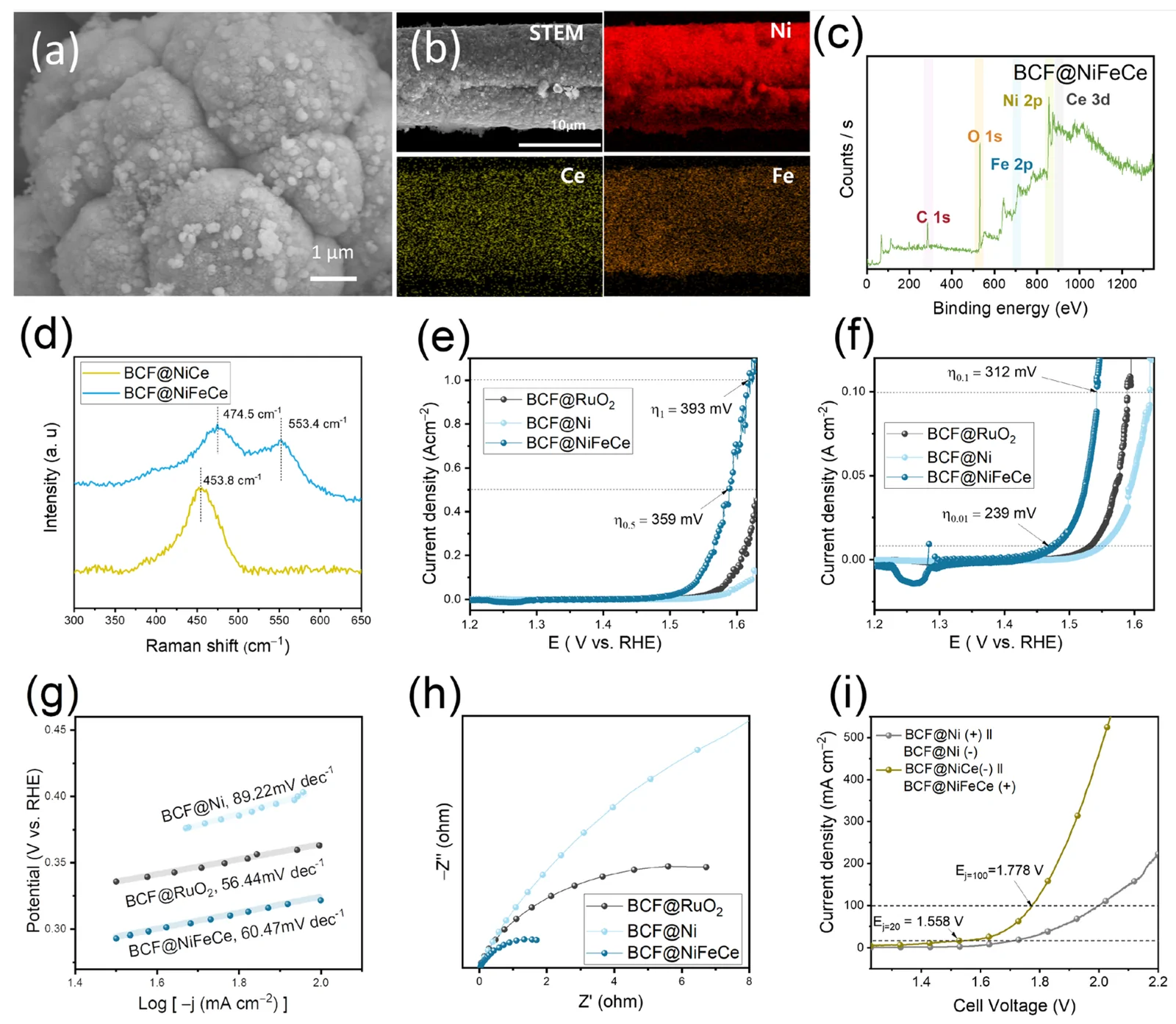
Characterization and OER performance of BCF@NiFeCe: (**a**) SEM image; (**b**) HAADF-STEM image and the corresponding EDX elemental maps (Ni, Ce, Fe); (**c**) XPS survey spectrum; (**d**) Raman spectra of BCF@NiCe and BCF@NiFeCe; (**e**) OER LSV curves of BCF@RuO_2_, BCF@Ni, and BCF@NiFeCe in 1 M KOH; (**f**) low-current-density region; (**g**) Tafel plots; (**h**) Nyquist plots; (**i**) two-electrode overall-water-splitting polarization curves of the BCF@NiCe(−) ∥ BCF@NiFeCe(+) couple compared with BCF@Ni(−) ∥ BCF@Ni(+).

Adding 5 mM Fe^3+^ to the identical bath converts the same one-pot process into a synthesis of OER anodes (a photograph of the as-prepared electrode is shown in Figure S12). SEM shows that BCF@NiFeCe retains the conformal fiber-wrapping architecture, with a finer, nanoparticle-decorated surface texture (Figure 6a), and STEM/EDX mapping confirms the homogeneous co-distribution of Ni, Ce, and Fe throughout the shell (Figure 6b). The XPS survey spectrum shows the additional Fe 2p signal (Figure 6c), and XRD reveals that the metallic framework now crystallizes as a NiFe alloy, with the (111), (200), and (220) reflections shifted relative to pure Ni (Figure S13). High-resolution Ni 2p, Fe 2p, and Ce 3d spectra (Figure S14) show mixed-valence Fe^2+^/Fe^3+^ together with the Ce^3+^/Ce^4+^ multiplet, i.e., the as-deposited anode already contains the redox-active constituents of the NiFe(-Ce) (oxy)hydroxide surface phase that forms in situ under anodic polarization [16,26]. Notably, the Ce 3d spectrum of BCF@NiFeCe is dominated by Ce^3+^ (≥~78%; Figure S14c), in sharp contrast to the Ce^4+^-dominant CeO_2−x_ of BCF@NiCe, indicating that, in the Fe-containing deposit, cerium is incorporated in a Ce(OH)_3_-like hydroxide environment consistent with the hydroxide-mediated co-precipitation pathway [16].

The OER activity (Figure 6e,f) reflects this composition. BCF@NiFeCe requires overpotentials of 239 mV at 10 mA cm^−2^, 312 mV at 0.1 A cm^−2^, 359 mV at 0.5 A cm^−2^, and 393 mV at 1 A cm^−2^. The RuO_2_ benchmark on the same fabric (BCF@RuO_2_) is competitive only at the lowest current densities: above ~0.1 A cm^−2^ the PGM-free anode delivers the higher current at any potential. The Tafel slope of BCF@NiFeCe (60 mV dec^−1^) approaches that of BCF@RuO_2_ (56 mV dec^−1^) and is far below that of BCF@Ni (89 mV dec^−1^) (Figure 6g), and EIS again shows the smallest charge-transfer resistance for the ternary catalyst (Figure 6h). The reverse-scan protocol used here excludes the contribution of the Ni^2+^/Ni^3+^ redox wave visible near 1.3 V vs. the reversible hydrogen electrode (RHE) (Figure 6f), so these metrics represent steady OER currents. The role of Ce is consistent with its reported functions in Ni–Fe (oxy)hydroxides—modulating the electronic structure of the Fe/Ni active sites and stabilizing the active phase through its facile Ce^3+^/Ce^4+^ cycling [16,17,27,28,29,30,31]—while the in situ transformation of the alloy surface into the active NiOOH/FeOOH layer under OER conditions is well documented for NiFe electrodes [26].

Raman spectroscopy (Figure 6d) independently corroborates the distinct Ce chemistries of the two catalysts. BCF@NiCe exhibits a single broad band at 453.8 cm^−1^, assigned to the F_2_g mode of fluorite CeO_2_ that is red-shifted and broadened relative to bulk CeO_2_ (464 cm^−1^)—the characteristic signature of nanocrystalline, oxygen-vacancy-rich CeO_2−x_ [18,19]—in quantitative agreement with the lattice expansion observed by HRTEM (Figure 2e) and the Ce^3+^ population from XPS (Figure 2i). An assignment to nickel hydroxide is excluded by the absence of the α-Ni(OH)_2_ companion mode at ~497 cm^−1^ and by the breadth of the band. BCF@NiFeCe, prepared by the same chemistry, shows an entirely different spectral pattern—broad bands at 474.5 and 553.4 cm^−1^, attributable to Ni–Fe (oxy)hydroxide surface species [16]—with no discernible F_2_g band, mirroring its Ce^3+^-dominant XPS and confirming that the 453.8 cm^−1^ band is specific to the CeO_2−x_ phase of the HER catalyst. The long-term durability of the optimized electrodes under constant-current operation at 1 A cm^−2^ is presented in Section 2.4 for the HER cathode (Figure 5a) and in Figure S15 for the OER anode: neither trace shows the progressive drift associated with catalyst detachment or dissolution, corroborating the mechanical robustness expected from the binder-free, conformally grown shells and consistent with the strong-adhesion rationale of high-current electrodeposition [9].

Finally, the two optimized electrodes were paired in a two-electrode alkaline water-splitting cell (Figure 6i). The BCF@NiCe(−) ∥ BCF@NiFeCe(+) couple delivers 20 mA cm^−2^ at a cell voltage of 1.558 V and 100 mA cm^−2^ at 1.778 V and substantially outperforms the symmetric BCF@Ni ∥ BCF@Ni cell over the entire range, demonstrating that the two products of the same one-pot bath directly assemble into a functional PGM-free electrolyzer operating in the practical two-electrode configuration. The Faradaic efficiency, measured by water displacement in a H-cell at 200 mA cm^−2^ (Figure S16), is 97–99% for H_2_ and 96–98% for O_2_, confirming that the measured currents correspond quantitatively to water splitting.

### 2.6. Generality of the One-Pot Platform

**Figure 7:**
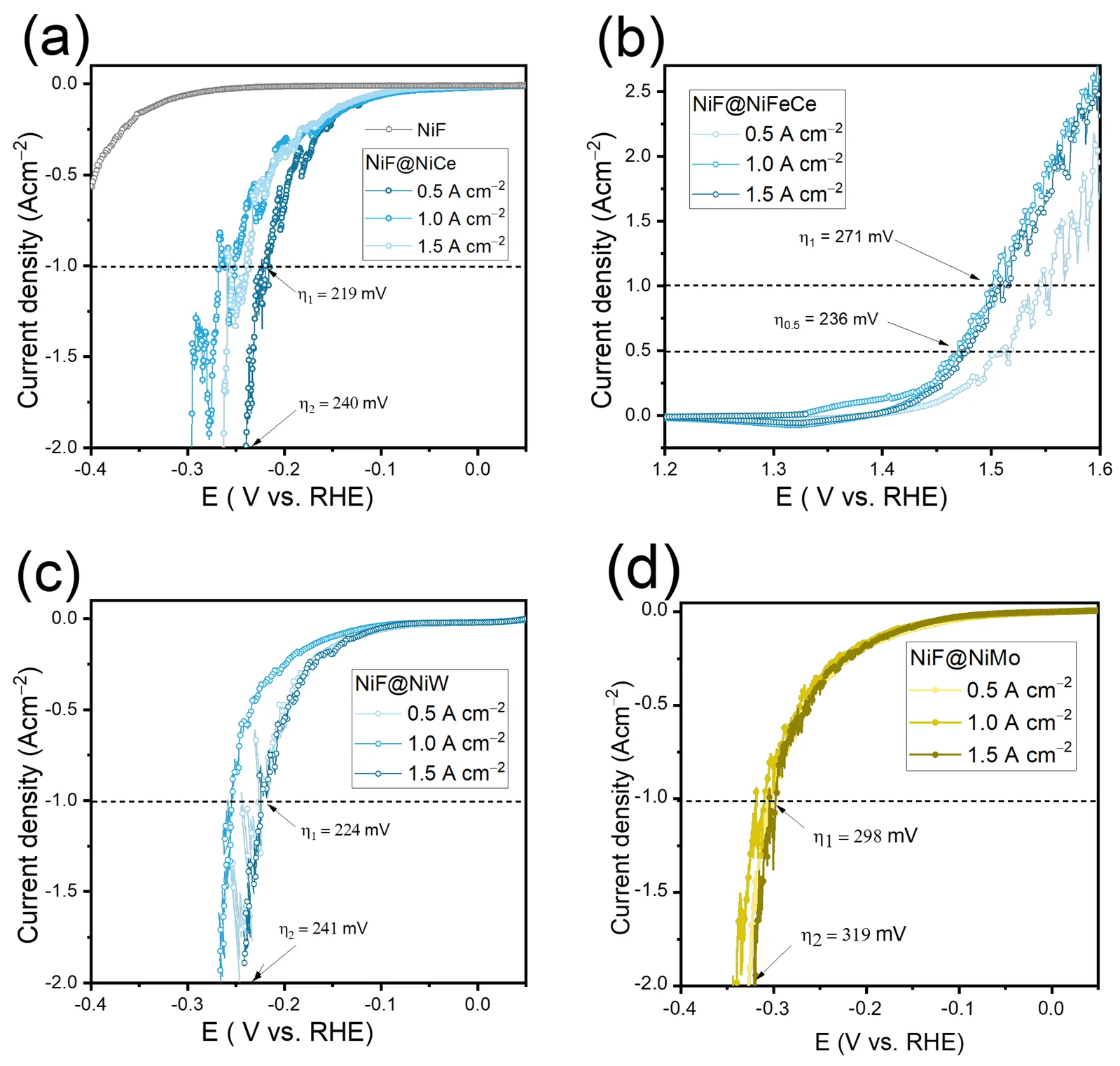
Generality of the one-pot platform on Ni foam (NiF): polarization curves of (**a**) NiF@NiCe (HER), (**b**) NiF@NiFeCe (OER), (**c**) NiF@NiW (HER), and (**d**) NiF@NiMo (HER), each prepared at deposition current densities of 0.5–1.5 A cm^−2^ and compared with bare NiF.

A central claim of this work is that the high-current NH_4_Cl deposition chemistry is a versatile route, in which the deposited catalyst is selected simply by the 5 mM secondary ion dissolved in the base bath. Figure 7 tests this claim along two axes: the substrate and the element. On nickel foam (NiF)—an industrially standard three-dimensional substrate—the identical 15 min galvanostatic protocol produces active catalysts in every case examined. NiF@NiCe reproduces the HER behavior observed on BCF, including the current-density dependence with an optimum near 1 A cm^−2^ (Figure 7a; low-current-density regions in Figure S17; η at 2 A cm^−2^ = 240 mV), and NiF@NiFeCe likewise reproduces the OER activity (Figure 7b). Replacing Ce with W or Mo yields NiF@NiW and NiF@NiMo, both of which are active HER catalysts, consistent with the extensive literature on Ni–W [32] and Ni–Mo [7,33,34,35,36] alkaline HER catalysts, whose overpotentials again vary systematically with the deposition current density (Figure 7c,d). The consistent ranking across compositions—deposits prepared near 1 A cm^−2^ outperform those prepared at lower or higher rates—indicates that the deposition-window physics identified in Section 2.2 is a property of the bath chemistry rather than of any particular metal couple. We expect this route to extend to transition-metal couples whose chloride/ammine complexes are stable in the concentrated NH_4_Cl bath and whose reduction potentials fall within the accessible deposition window; systems requiring markedly different complexation chemistry would need an adapted bath. We emphasize what this generality means in practice: with one stock bath, one power supply, and a shelf of common metal salts, both electrodes of an alkaline electrolyzer, on either a biomass-derived or a commercial substrate, are produced by the same fifteen-minute room-temperature process.

## 3. Materials and Methods

### 3.1. Materials

Nickel(II) chloride hexahydrate (NiCl_2_·6H_2_O), ammonium chloride (NH_4_Cl), cerium(III) chloride heptahydrate (CeCl_3_·7H_2_O), iron(III) nitrate nonahydrate (Fe(NO_3_)_3_·9H_2_O), sodium tungstate dihydrate (Na_2_WO_4_·2H_2_O), ammonium heptamolybdate tetrahydrate ((NH_4_)_6_Mo_7_O_24_·4H_2_O), potassium hydroxide (KOH), and hydrochloric acid (HCl) were purchased from Sigma-Aldrich (St. Louis, MO, USA) and used as received. A commercial disposable kitchen wipe made of natural bamboo-fiber pulp (daily bamboo dishcloth; imported by Linder Korea Co., Ltd., Bucheon-si, Republic of Korea) was used as the cellulose precursor for the carbon fabric substrate. Nickel foam (NiF; thickness 1.6 mm, 110 PPI; SJmaterial Co., Seoul, Republic of Korea) was used as an auxiliary deposition substrate. A commercial mixed-metal-oxide-coated titanium mesh (MMO (Ru–Ir oxide)/Ti dimensionally stable anode; TISTAR, purchased via AliExpress) was used as the counter electrode (anode) for electrodeposition. Deionized (DI) water (18.2 MΩ·cm) was used throughout.

### 3.2. Preparation of the Bamboo-Derived Carbon Fabric (BCF)

A low-cost, commercially available nonwoven wipe composed of natural bamboo cellulose fibers was selected as the carbon precursor, motivated by the rapid renewability of bamboo and the negligible cost of the mass-produced consumer product. Fabric sheets (27.5 × 27.5 cm) were carbonized at 1100 °C for 3 h under an Ar atmosphere (heating rate 5 °C min^−1^), yielding freestanding carbon fabrics of 16.5 × 16.5 cm (≈36% of the original area) that retained the fibrous network and mechanical flexibility of the pristine fabric. The bamboo-derived carbon fabric (hereafter BCF) was cut into 2 × 2 cm pieces and used directly as the deposition substrate without any binder, conductive additive, or surface treatment.

### 3.3. One-Pot High-Current-Density Electrodeposition

All catalyst layers were deposited from a common base bath consisting of 0.10 M NiCl_2_·6H_2_O (24 g L^−1^) and 2.0 M NH_4_Cl (107 g L^−1^) in DI water, in which the high-concentration NH_4_Cl served as the sole supporting electrolyte without any pH buffer or complexing agent, following the deposition chemistry recently reported by Serban et al. [10] and extending it to new metal combinations and substrates. The secondary metal precursors were added to the base bath at a fixed concentration of 5 mM on a metal basis to allow direct comparison among the different Ni–M systems: 1.86 g L^−1^ CeCl_3_·7H_2_O (5 mM Ce) for Ni–Ce (denoted BCF@NiCe); 1.86 g L^−1^ CeCl_3_·7H_2_O plus 2.02 g L^−1^ Fe(NO_3_)_3_·9H_2_O (5 mM Ce + 5 mM Fe) for Ni–Fe–Ce (denoted BCF@NiFeCe); 1.65 g L^−1^ Na_2_WO_4_·2H_2_O (5 mM W) for Ni–W; and 0.88 g L^−1^ (NH_4_)_6_Mo_7_O_24_·4H_2_O (0.71 mM heptamolybdate, corresponding to 5 mM Mo) for Ni–Mo. A Ni-only control (BCF@Ni) was prepared from the additive-free base bath by conventional low-current electroplating (0.25 A cm^−2^ for 15 min), providing a plain Ni surface on the same fabric that plays the role of a conventional nickel substrate (cf. Ni foam or Ni felt).

Electrodeposition was performed in a two-electrode configuration driven by a programmable DC power supply (RD6030-Max, RIDEN, Hangzhou, China) operated in constant-current (galvanostatic) mode, with the BCF (2 × 2 cm) as the working electrode (cathode) and the MMO/Ti mesh as the anode. A silicone mesh (2 × 2 cm) was placed between the electrodes as a porous spacer, preventing short-circuiting while allowing ionic transport; using the spacer as an uncut single sheet was found to be essential for stable, noise-free deposition on BCF. Each deposition used a 150 mL bath in a rectangular silicone mold (10 × 10 cm), and separate baths and power supplies were used for each composition to avoid cross-contamination. Depositions were carried out at room temperature without stirring.

The deposition current density (based on the geometric area of 4 cm^2^) was systematically varied at 0.25, 0.5, 0.75, 1.0, 1.5, 2.0, and 3.0 A cm^−2^ (total applied currents of 1–12 A) at a fixed deposition time of 15 min. The optimal condition was determined to be 1 A cm^−2^ (4 A) for 15 min based on the HER activity of BCF@NiCe; unless otherwise noted, all samples discussed hereafter were prepared under this condition. For the generality tests, catalyst layers were deposited on Ni foam (1 × 2 cm; pretreated by brief immersion in 6 M HCl, rinsed twice with DI water, and used immediately without drying) under the same fixed-time protocol at 0.5–1.5 A cm^−2^. For the time- and concentration-variation studies, deposits were additionally prepared for 5–60 min and from baths containing 2.5 or 10 mM CeCl_3_ (denoted BCF@NiCe-2.5 and BCF@NiCe-10, respectively). Catalyst loadings were determined gravimetrically as the electrode mass gain after deposition (identical drying protocol before and after weighing), and apparent current efficiencies were calculated from Faraday’s law assuming two-electron reduction of Ni^2+^. The working-electrode potential during galvanostatic deposition was recorded separately in a three-electrode configuration by inserting a Hg/HgO reference electrode, verified against a calibrated reference before and after use, into the deposition bath.

### 3.4. Materials Characterization

Surface morphologies were examined by field-emission scanning electron microscopy (FE-SEM; Hitachi S-4700 and SU-8600, Tokyo, Japan) equipped with energy-dispersive X-ray spectroscopy (EDX) for elemental mapping. Phase identification was conducted via X-ray diffraction (XRD; X’Pert PRO, PANalytical, Almelo, The Netherlands, Cu Kα radiation), and surface chemical states were analyzed using X-ray photoelectron spectroscopy (XPS; K-ALPHA, Thermo Fisher Scientific, Waltham, MA, USA) with binding energies calibrated to the C 1s peak at 284.8 eV. The Ni 2p spectra were deconvoluted into Ni^0^, Ni^2+^, and satellite components on a Shirley background. The Ce 3d spectra were deconvoluted into the standard ten-component set (two Ce^3+^ and three Ce^4+^ spin–orbit doublets, conventional v/u notation) with a common spin–orbit splitting and a fixed 3d_3_/_2_/3d_5_/_2_ area ratio of 2:3; owing to the overlap of the Ce 3d region with Ni LMM Auger emission, a spline background anchored at the inter-peak minima was employed. TEM, HRTEM, and scanning TEM (STEM)-EDX analyses, including high-angle annular dark-field (HAADF) imaging, were performed on a 200 kV transmission electron microscope (Talos F200X G2, Thermo Fisher Scientific, Waltham, MA, USA) equipped with a Ceta 16M camera; the catalyst was detached from the fabric, dispersed in ethanol by sonication, and drop-cast onto TEM grids, and lattice spacings were determined by fast Fourier transform (FFT) analysis of the calibrated images. Raman spectra were acquired using a confocal Raman spectrometer (LabRAM HR Evolution, Horiba, Kyoto, Japan) with 532 nm laser excitation at low laser power to avoid local heating. The catalyst loadings were determined gravimetrically, and the elemental compositions of the deposits were quantified by area-averaged SEM-EDX (*n* ≥ 5 regions). In situ bubble imaging during HER at 0.2 A cm^−2^ was performed with a high-speed camera; the near-surface bubble populations were segmented by a threshold–fill-holes–watershed procedure with a circularity filter of 0.7–1.0 (ImageJ 1.54, National Institutes of Health, Bethesda, MD, USA) and are reported as area-equivalent circular diameters. Surface wettability was assessed by recording the absorption of DI water droplets placed on the electrode surface with a high-speed camera.

### 3.5. Electrochemical Evaluation

All electrochemical tests were conducted using a ZIVE BCP2 potentiostat (WONATECH, Seoul, Republic of Korea). Three-electrode measurements were performed in 1 M KOH using a sample size of 1 × 2 cm^2^, with a graphite rod as the counter electrode and a Hg/HgO (1 M NaOH) reference electrode. All potentials were converted to the reversible hydrogen electrode (RHE) scale using H_2_-saturated calibration. HER activity was assessed by linear sweep voltammetry (LSV) with a forward scan (1 mV s^−1^), while OER was evaluated in reverse scan mode to minimize the contribution of the Ni redox features. Benchmark electrodes were prepared by spraying commercial Pt/C (for HER) and RuO_2_ (for OER) onto the BCF at a loading of 1.0 mg cm^−2^ (denoted BCF@Pt/C and BCF@RuO_2_) and were measured under identical conditions. Electrochemical impedance spectroscopy (EIS) was conducted at an overpotential of 200 mV using a 10 mV perturbation over a frequency range of 0.1 Hz to 1 MHz. Long-term durability was evaluated by chronopotentiometry in the same three-electrode configuration at a constant current density of 1 A cm^−2^ for 200 h for BCF@NiCe (HER) and BCF@NiFeCe (OER), respectively, with periodic replenishment of DI water to compensate electrolyte evaporation. Prior to data collection, the electrodes were conditioned by 30 CV cycles at 50 mV s^−1^ between 0 to −0.25 V vs. RHE for the HER electrodes and 1.2 to 1.6 V vs. RHE for the OER electrodes until reproducible voltammograms were obtained. Double-layer capacitances were determined from CVs recorded in a 0.10 V non-Faradaic window at scan rates of 20–100 mV s^−1^, using the final cycle at each scan rate; ECSAs were estimated assuming a specific capacitance of 40 µF cm^−2^ [24]. Faradaic efficiencies were measured in a H-cell by the water-displacement method at 200 mA cm^−2^, comparing the collected H_2_ and O_2_ volumes with the theoretical values from Faraday’s law. Two-electrode overall-water-splitting measurements paired BCF@NiCe (cathode) with BCF@NiFeCe (anode) in the same electrolyte. Mechanical robustness was evaluated by ultrasonicating the electrodes in deionized water (40 kHz, 60 min) and comparing them with a binder-coated control (Pt/C + Nafion on BCF).

## 4. Conclusions

We have established a one-pot, high-current-density electrodeposition platform that converts a mass-produced bamboo-cellulose wipe into high-performance, self-supported electrodes for alkaline water splitting. A single NiCl_2_/NH_4_Cl bath, differentiated only by 5 mM of a selectable secondary metal ion, produces conformal polycrystalline catalyst shells on the individual carbon fibers within fifteen minutes at room temperature. The fixed-time screening identifies a deposition window centered at 1 A cm^−2^—far above conventional plating practice—as essential to compact, adherent, and active deposits. The resulting BCF@NiCe cathode couples an intrinsically enhanced Ni/CeO_2−x_ interface—nanocrystalline, oxygen-vacancy-rich ceria intermixed with metallic Ni, verified by HRTEM, Raman, and XPS and quantified by ECSA-normalized activities—with a superhydrophilic, fine-bubble-releasing fibrous architecture to rival and, at ampere-level current densities, surpass Pt/C for the HER, while the Fe-co-deposited BCF@NiFeCe anode outperforms RuO_2_ for the OER above 0.1 A cm^−2^. Both electrodes endure 200 h of continuous operation, with the nanoscale Ni/CeO_2−x_ structure, the oxygen-vacancy population, and the surface chemical states fully preserved, and the identical protocol extends to Ni–W and Ni–Mo on nickel foam. By uniting a consumer-goods-derived substrate, an additive-free bath, and a DC power supply, this platform reduces the fabrication of ampere-level water-splitting electrodes to a routine, scalable operation, and we anticipate its extension to further Ni–M combinations and to device-level AEMWE integration.

## Data Availability

The data supporting this study are available from the corresponding author upon reasonable request.

## Supplementary Materials

The following supporting information can be downloaded at https://www.mdpi.com/article/10.3390/molecules31172969/s1. Figure S1: SEM image of BCF@NiCe prepared under the optimized galvanostatic condition (1 A cm^−2^, 15 min), showing the nodular Ni–Ce deposit uniformly enveloping an individual carbon fiber; Figure S2: SEM image and the corresponding EDX elemental maps of BCF@Ni prepared by low-current Ni electroplating (0.25 A cm^−2^, 15 min), showing a conformal Ni shell around the individual carbon fibers; Figure S3: SEM image and the corresponding EDS elemental maps (Ni, O, and Ce) of a single BCF@NiCe fiber, showing the uniform co-distribution of Ni, Ce, and O throughout the deposited shell; Figure S4: High-resolution Ni 2p XPS spectrum of BCF@Ni with deconvolution into metallic Ni^0^, Ni^2+^, and satellite components (Shirley background); Figure S5: Chronopotentiometric profiles recorded during galvanostatic electrodeposition at 0.50, 0.75, and 1.00 A cm^−2^ in a three-electrode configuration with a Hg/HgO reference electrode, showing stable quasi-steady deposition potentials without passivation-type excursions; Figure S6: HER polarization curves of (a) BCF@NiCe deposited at 1.0 A cm^−2^ for 5–60 min and (b) BCF@NiCe-2.5 and BCF@NiCe-10, prepared from plating baths containing 2.5 and 10 mM CeCl, respectively, showing the optimization of the deposition time and the Ce precursor concentration; Figure S7: Nyquist plots of BCF@Pt/C, BCF@Ni, and BCF@NiCe measured in the HER region at an overpotential of 200 mV in 1 M KOH; Figure S8: Cyclic voltammograms in the non-Faradaic potential region at scan rates of 20–100 mV s^−1^ for (a) BCF@Ni, (b) BCF@NiCe, and (c) BCF@NiFeCe, and (d) the corresponding Δj/2 versus scan rate plots, yielding double-layer capacitances of 1.10, 9.85, and 9.11 mF cm^−2^, respectively; Figure S9: ECSA-normalized polarization curves of (a) BCF@Ni and BCF@NiCe for the HER and (b) BCF@Ni and BCF@NiFeCe for the OER. ECSA values were obtained from the double-layer capacitances assuming a specific capacitance of 40 µF cm^−2^; Figure S10: SEM image of BCF@NiCe after the 200 h HER durability test, showing that the deposit morphology and adhesion are preserved; Figure S11: Photographs of BCF@NiCe and a binder-coated control electrode (Pt/C + Nafion on BCF) (a) before and (b) after ultrasonication in deionized water for 60 min. Severe catalyst shedding (darkened solution) is observed only for the binder-coated control, whereas the directly electrodeposited BCF@NiCe remains intact; Figure S12: Photograph of the BCF@NiFeCe electrode (2 × 2 cm) prepared under the optimized galvanostatic condition (1 A cm^−2^, 15 min); Figure S13: XRD pattern of BCF@NiFeCe, showing the (111), (200), and (220) reflections of the NiFe alloy phase; Figure S14: High-resolution XPS spectra of BCF@NiFeCe with deconvolution: (a) Ni 2p, (b) Fe 2p, and (c) Ce 3d; Figure S15: Chronopotentiometric stability of BCF@NiFeCe (OER) at a constant current density of 1 A cm^−2^ in 1 M KOH for 200 h; Figure S16: Faradaic efficiency measured by the water-displacement method in an H-cell at 200 mA cm^−2^: evolved H_2_ and O_2_ volumes as a function of time and the corresponding Faradaic efficiencies of ~97–99% (H_2_) and ~96–98% (O_2_); Figure S17: Extension of the NH_4_Cl-assisted high-current-density electrodeposition to a Ni felt (NiF) substrate: polarization curves of NiF@NiCe, NiF@NiW, and NiF@NiMo (HER) and NiF@NiFeCe (OER) deposited at 0.5–1.5 A cm^−2^, compared with bare NiF.
