# Supplementary material for: One-Pot High-Current-Density Electrodeposition of Ni–Ce and Ni–Fe–Ce Catalysts on Bamboo-Derived Carbon Fabric for Alkaline Water Splitting
Source: Molecules. 2026 Aug 25;31(17):2969. doi: 10.3390/molecules31172969 (PMC13567567; doi:10.3390/molecules31172969)
Supplement: Supplementary file 1 [file molecules-31-02969-s001.zip › molecules-4496946-supplementary.pdf]

Supplementary Materials

One-Pot High-Current-Density Electrodeposition of Ni–Ce and Ni–Fe–Ce Catalysts on Bamboo-Derived Carbon Fabric for Alkaline Water Splitting

Sun-Woo Lee, Sunghoon Ahn*

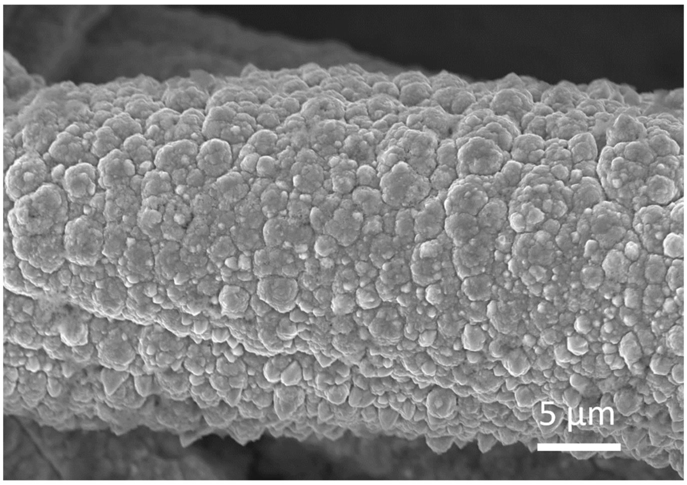

Figure S1. SEM image of BCF@NiCe prepared under the optimized galvanostatic condition (1 A cm^{-2}, 15 min), showing the nodular Ni–Ce deposit uniformly enveloping an individual carbon fiber.

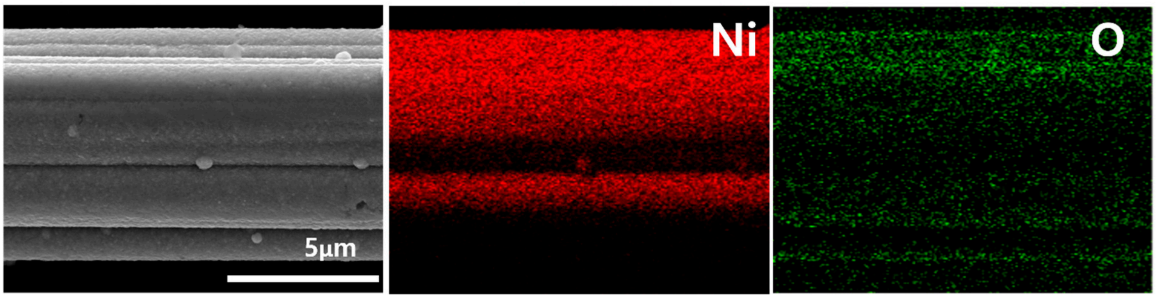

Figure S2. SEM image and the corresponding EDX elemental maps of BCF@Ni prepared by low-current Ni electroplating (0.25 A cm^{-2}, 15 min), showing a conformal Ni shell around the individual carbon fibers.

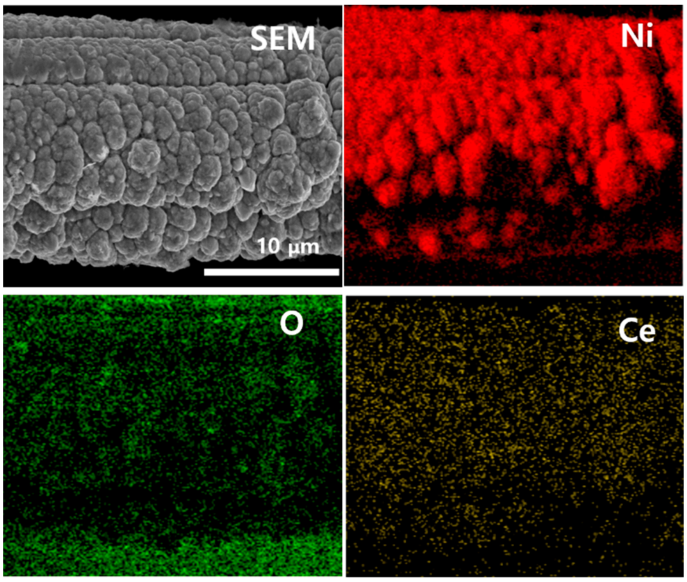

Figure S3. SEM image and the corresponding EDS elemental maps (Ni, O, and Ce) of a single BCF@NiCe fiber, showing the uniform co-distribution of Ni, Ce, and O throughout the deposited shell.

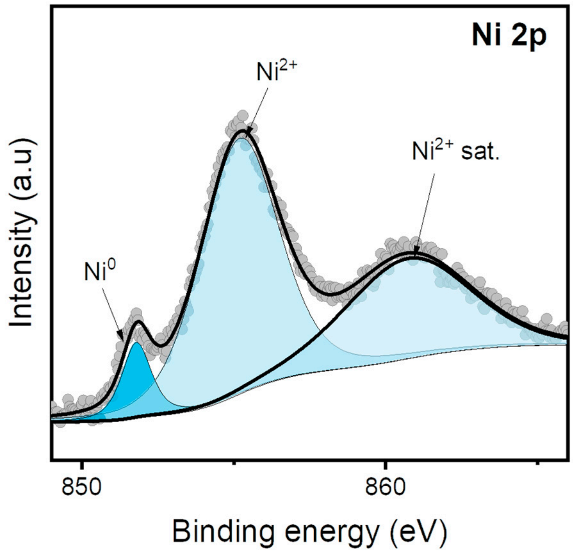

Figure S4. High-resolution Ni 2p XPS spectrum of BCF@Ni with deconvolution into metallic Ni^0, Ni^{2+}, and satellite components (Shirley background).

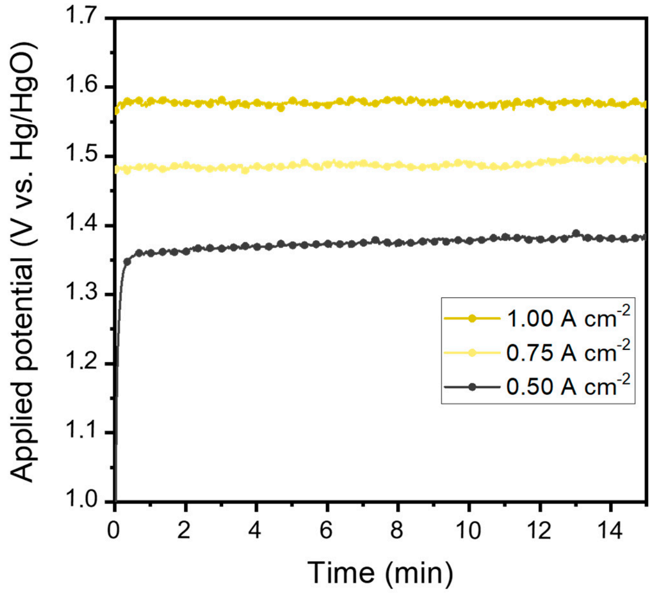

Figure S5. Chronopotentiometric profiles recorded during galvanostatic electrodeposition at 0.50, 0.75, and 1.00 A cm^{-2} in a three-electrode configuration with a Hg/HgO reference electrode, showing stable quasi-steady deposition potentials without passivation-type excursions.

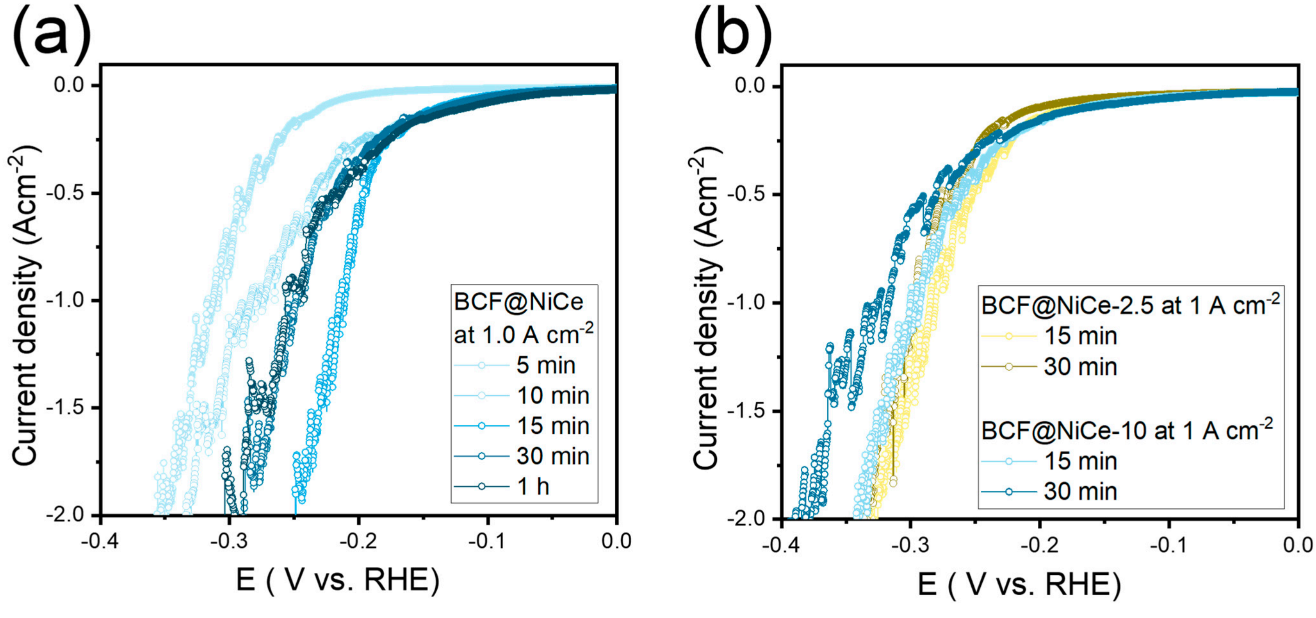

Figure S6. HER polarization curves of (a) BCF@NiCe deposited at 1.0 A cm^{-2} for 5–60 min and (b) BCF@NiCe-2.5 and BCF@NiCe-10, prepared from plating baths containing 2.5 and 10 mM CeCl, respectively, showing the optimization of the deposition time and the Ce precursor concentration.

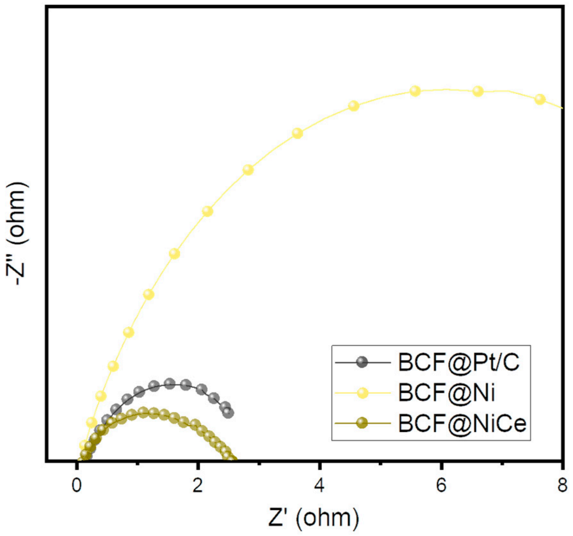

Figure S7. Nyquist plots of BCF@Pt/C, BCF@Ni, and BCF@NiCe measured in the HER region at an overpotential of 200 mV in 1 M KOH.

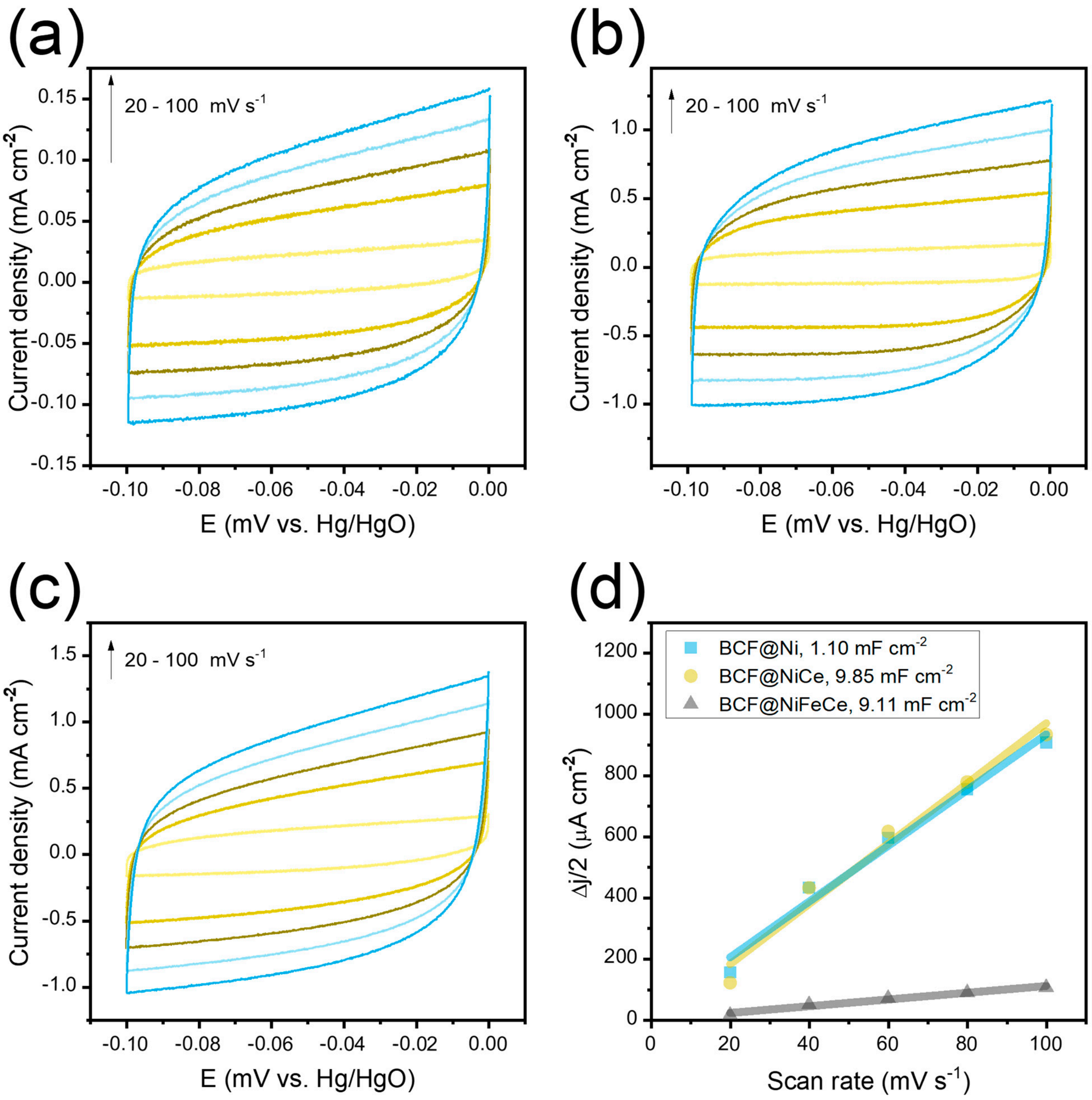

Figure S8. Cyclic voltammograms in the non-Faradaic potential region at scan rates of 20–100 mV s^{-1} for (a) BCF@Ni, (b) BCF@NiCe, and (c) BCF@NiFeCe, and (d) the corresponding Δj/2 versus scan rate plots, yielding double-layer capacitances of 1.10, 9.85, and 9.11 mF cm^{-2}, respectively.

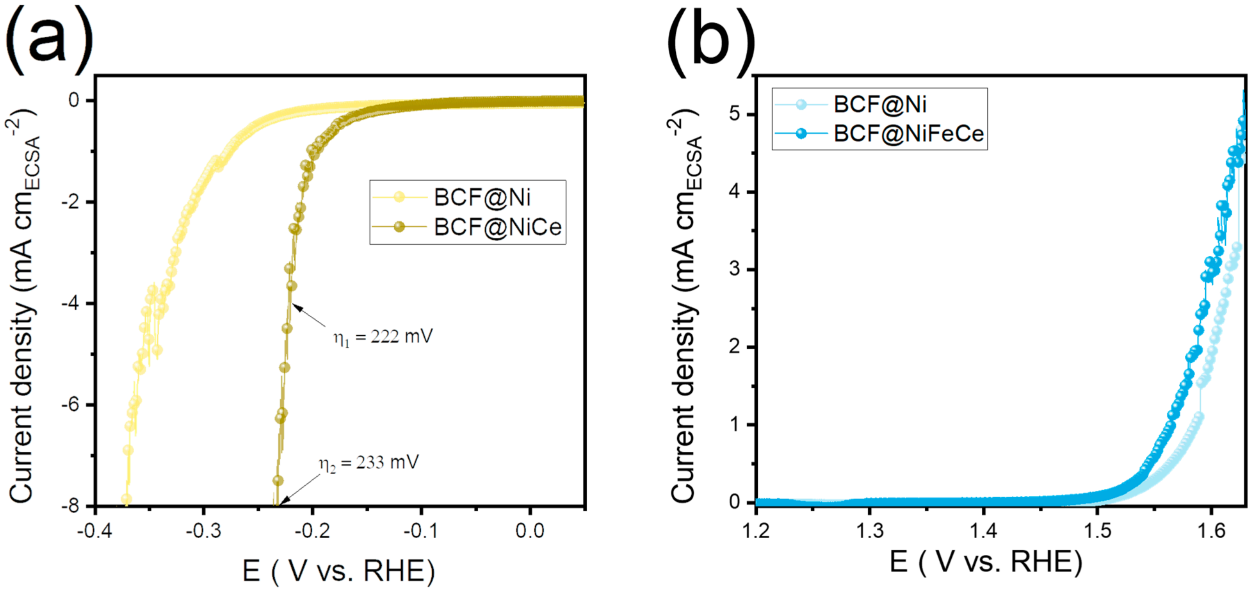

Figure S9. ECSA-normalized polarization curves of (a) BCF@Ni and BCF@NiCe for the HER and (b) BCF@Ni and BCF@NiFeCe for the OER. ECSA values were obtained from the double-layer capacitances assuming a specific capacitance of 40 μF cm^{-2}.

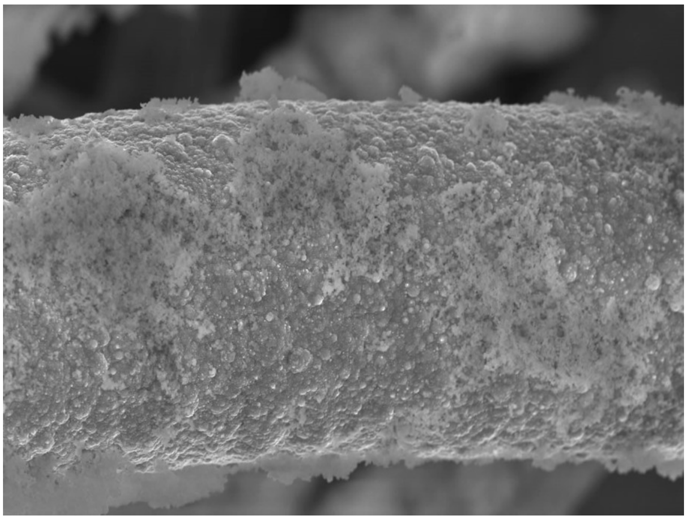

Figure S10. SEM image of BCF@NiCe after the 200 h HER durability test, showing that the deposit morphology and adhesion are preserved.

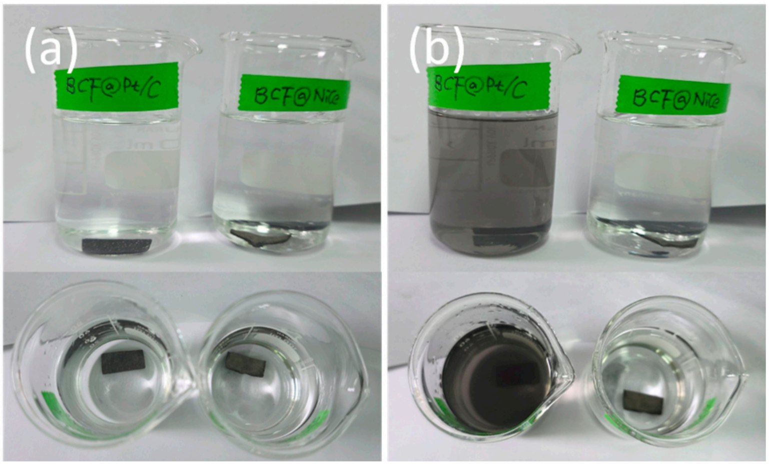

Figure S11. Photographs of BCF@NiCe and a binder-coated control electrode (Pt/C + Nafion on BCF) (a) before and (b) after ultrasonication in deionized water for 60 min. Severe catalyst shedding (darkened solution) is observed only for the binder-coated control, whereas the directly electrodeposited BCF@NiCe remains intact.

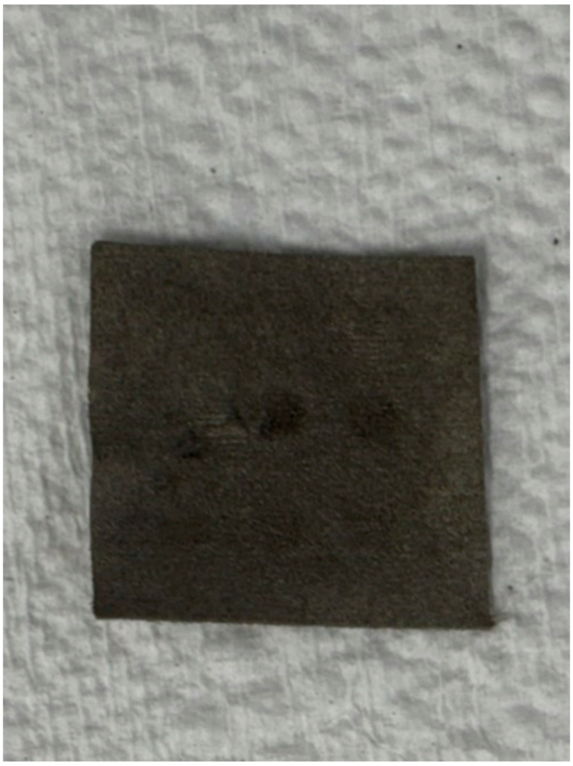

Figure S12. Photograph of the BCF@NiFeCe electrode (2 × 2 cm) prepared under the optimized galvanostatic condition (1 A cm^{-2}, 15 min).

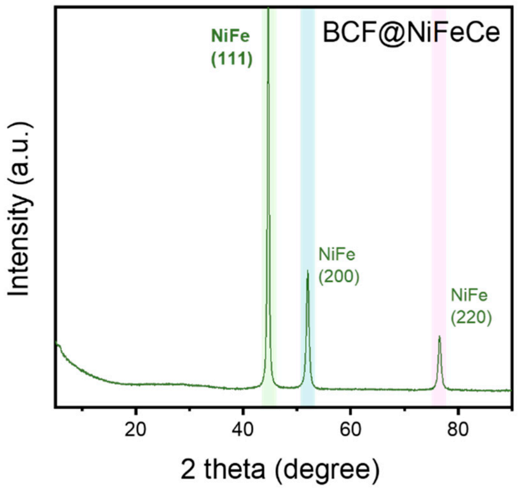

Figure S13. XRD pattern of BCF@NiFeCe, showing the (111), (200), and (220) reflections of the NiFe alloy phase.

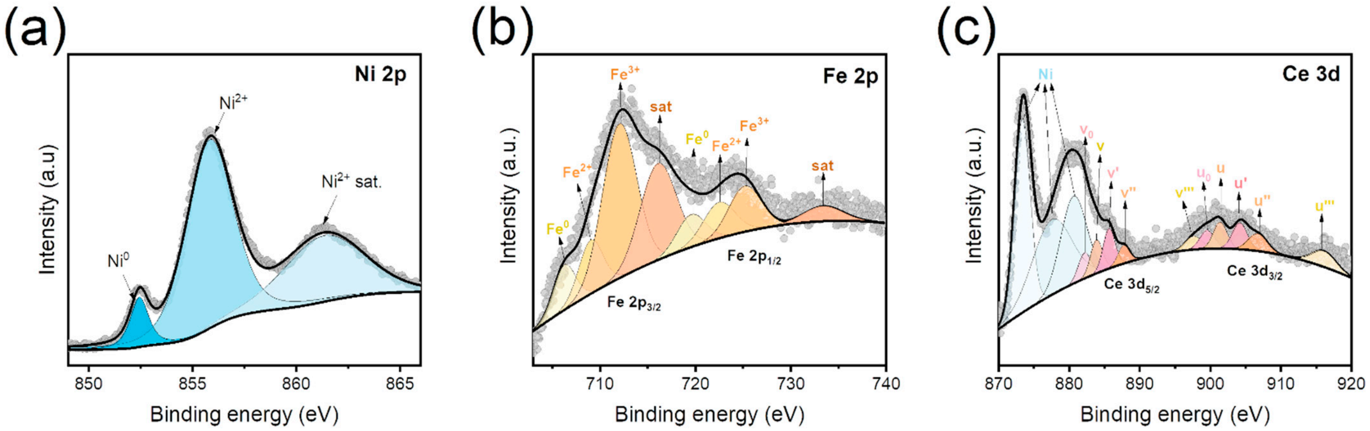

Figure S14. High-resolution XPS spectra of BCF@NiFeCe with deconvolution: (a) Ni 2p, (b) Fe 2p, and (c) Ce 3d.

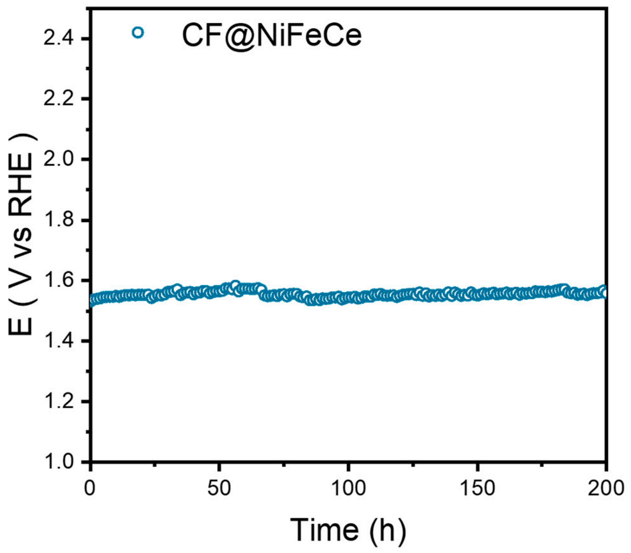

Figure S15. Chronopotentiometric stability of BCF@NiFeCe (OER) at a constant current density of 1 A cm^{-2} in 1 M KOH for 200 h.

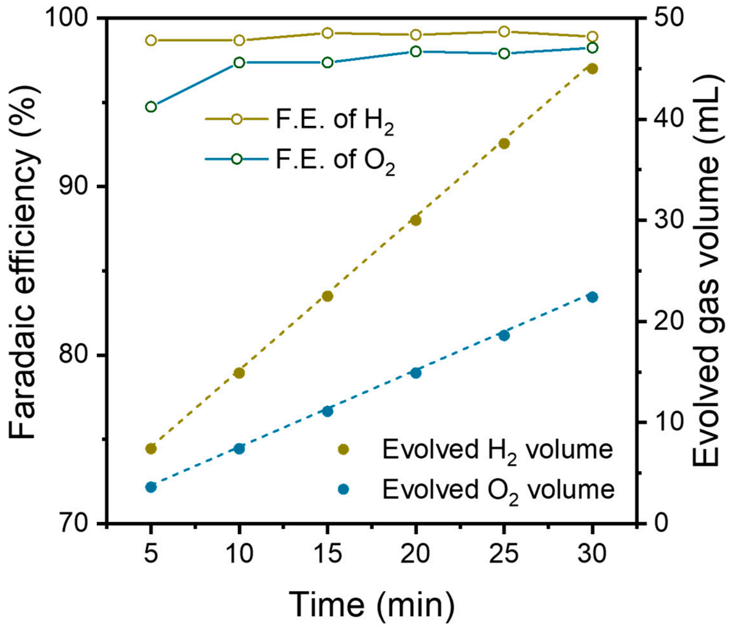

Figure S16. Faradaic efficiency measured by the water-displacement method in an H-cell at 200 mA cm^{-2}: evolved H_2 and O_2 volumes as a function of time and the corresponding Faradaic efficiencies of ~97–99% (H_2) and ~96–98% (O_2).

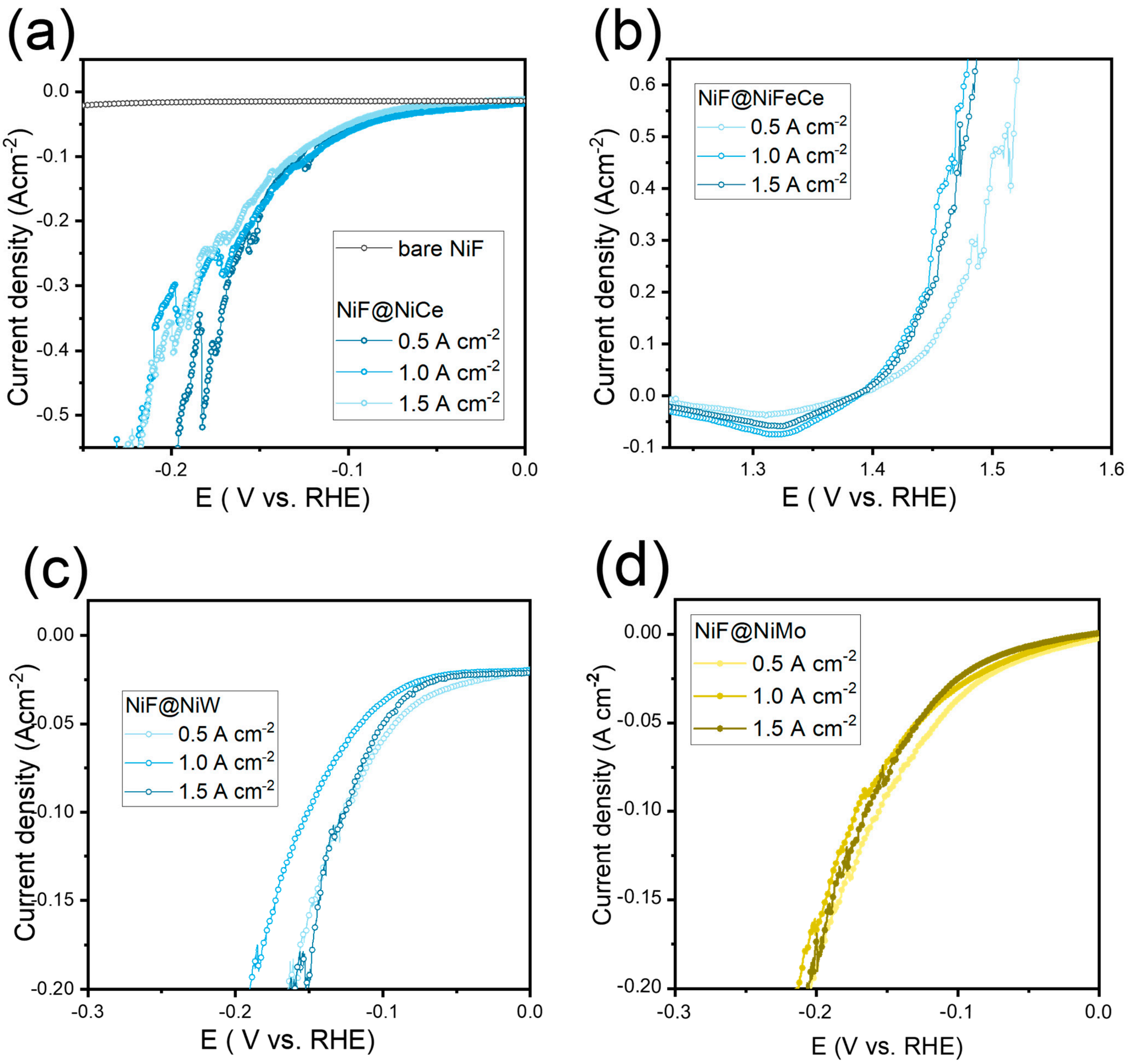

Figure S17. Extension of the NH_4 Cl-assisted high-current-density electrodeposition to a Ni felt (NiF) substrate: polarization curves of NiF@NiCe, NiF@NiW, and NiF@NiMo (HER) and NiF@NiFeCe (OER) deposited at 0.5–1.5 A cm^{-2}, compared with bare NiF.
